# Titanium as a Beneficial Element for Crop Production

**DOI:** 10.3389/fpls.2017.00597

**Published:** 2017-04-25

**Authors:** Shiheng Lyu, Xiangying Wei, Jianjun Chen, Cun Wang, Xiaoming Wang, Dongming Pan

**Affiliations:** ^1^College of Horticulture, Fujian Agriculture and Forestry UniversityFuzhou, China; ^2^Department of Environmental Horticulture, Mid-Florida Research and Education Center, Institute of Food and Agricultural Sciences, University of FloridaApopka, FL, USA; ^3^Tropical Crops Genetic Resources Institute, Chinese Academy of Tropical Agricultural SciencesDanzhou, China; ^4^Hunan Key Laboratory for Breeding of Clonally Propagated Forest Trees, Hunan Academy of ForestryChangsha, China

**Keywords:** beneficial elements, ferric chelate reductase, ferritins, iron, metal transporter, nano-TiO_2_ particles (TiO_2_NPs), titanium

## Abstract

Titanium (Ti) is considered a beneficial element for plant growth. Ti applied via roots or leaves at low concentrations has been documented to improve crop performance through stimulating the activity of certain enzymes, enhancing chlorophyll content and photosynthesis, promoting nutrient uptake, strengthening stress tolerance, and improving crop yield and quality. Commercial fertilizers containing Ti, such as Tytanit and Mg-Titanit, have been used as biostimulants for improving crop production; however, mechanisms underlying the beneficial effects still remain unclear. In this article, we propose that the beneficial roles Ti plays in plants lie in its interaction with other nutrient elements primarily iron (Fe). Fe and Ti have synergistic and antagonistic relationships. When plants experience Fe deficiency, Ti helps induce the expression of genes related to Fe acquisition, thereby enhancing Fe uptake and utilization and subsequently improving plant growth. Plants may have proteins that either specifically or nonspecifically bind with Ti. When Ti concentration is high in plants, Ti competes with Fe for ligands or proteins. The competition could be severe, resulting in Ti phytotoxicity. As a result, the beneficial effects of Ti become more pronounced during the time when plants experience low or deficient Fe supply.

## Introduction

Titanium (Ti), which has an atomic number 22 and atomic weight 47.88, is a transition element belonging to Group 4 (IVB) in the middle of the Periodical Table. It is the ninth most abundant element in the earth's crust and makes up about 0.25% by moles and 0.57% by weight of the crust (Buettner and Valentine, 2012). Ti is the second most abundant transition metal, after iron (Fe), and the elemental abundance of Ti is about 5 times less than Fe and 100 times greater than copper (Cu). Ti exhibits oxidation states of Ti^2+^, Ti^3+^ (titanous), and Ti^4+^ (titanic), of which Ti^2+^ and Ti^3+^ are unstable, while Ti^4+^ is the most stable ion. The most important compound is TiO_2_, which is mainly used in paints. TiCl_4_ is water soluble but is highly volatile and forms spectacular opaque clouds upon contact with humid air. Ti ascorbate is a synthesized compound which is soluble in water and stable up to pH 8.0.

The mineral sources of Ti include anatase, rutile, and brookite, each encompassing about 95% TiO_2_ as well as ilmenite (FeOTiO_3_) comprising 40–65% TiO_2_ and leucoxene (Fe_2_O_3_ nTiO_3_) containing more than 65% TiO_2_ (Zhang et al., 2011). These minerals are generally not soluble; thus Ti has been conventionally considered to be inert in the environment. Increasing evidence in the literature, however, suggests that Ti is mobile in rocks under weathering conditions (Kaup and Carter, 1987; Du et al., 2012). Ti may be mobile at the centimeter scale as well as at the profile scale under strong tropical weathering conditions (Cornu et al., 1999). Higher Ti contents occur in tropical soils, particularly in lateritic soils and laterites, such as 15% in Hawaii soils (Sherman, 1952); 15% in Norfolk Island soils (Hutton and Stephens, 1956), and 3.4% in Australian soils (Stace et al., 1968). Ti in surface soils worldwide ranges from 0.02 to 2.4% with a mean of 0.33%; Ti in soil solutions is about 30 mg L^−1^ (Kabata-Pendias and Mukherjee, 2007). Ti in river waters ranges from 0.02 to 2.3 μg L^−1^, and the worldwide average is estimated to be 0.49 μg L^−1^ (Kabata-Pendias and Pendias, 2001). Drinking waters in the US contain Ti from 0.5 to 15 μg L^−1^ (Anke and Seifert, 2004). Ti also exists in the atmosphere with global median values of 7 ng m^−3^ in the remote regions (away from anthropogenic releases) and 85 ng m^−3^ in polluted zones. Ti concentrations in the air of the US vary from 10 to 100 ng m^−3^ and can increase up to ≤1,000 ng m^−3^ in industrial regions (Kabata-Pendias and Mukherjee, 2007).

Titanium dioxide nanoparticles (TiO_2_NPs) are another form of Ti in the environment. TiO_2_NPs are produced worldwide at an estimated 88,000 t per year (Keller et al., 2013) and are utilized widely in the cosmetic, food, painting, and plastic industries. Due to their photoprotective and photocatalytic roles, TiO_2_NPs are also used for plant protection and environmental remediation. It is estimated that the concentrations of TiO_2_NPs in soils could reach 0.13 μg kg^−1^ yr^−1^ in Europe, and TiO_2_NPs in soils amended with sewage could be much higher up to 1,200 μg kg^−1^ yr^−1^ (Sun et al., 2014). With the increased exploration of nanomaterials for novel commercial applications, TiO_2_NPs in soils could increase from 3 to more than 5,000 μg kg^−1^ yr^−1^ (Gogos et al., 2012; Kah et al., 2013).

## Ti in higher plants

**Figure 1:**
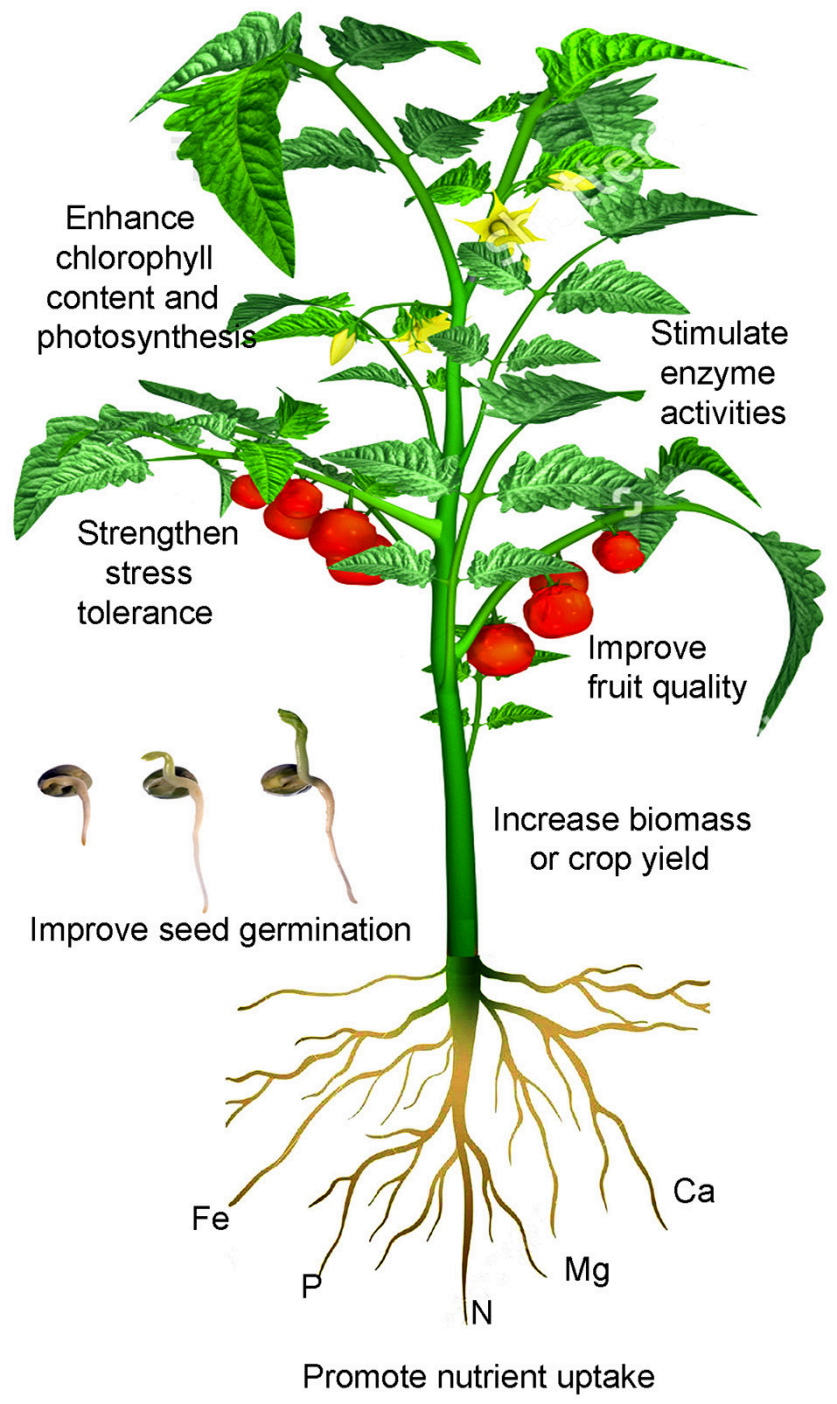
**A schematic illustration of Ti effects on crop performance**. Ti applied via roots or leaves at appropriately low concentrations has been shown to promote seed germination, enhance root uptake of other nutrient elements, stimulate the activity of some enzymes, increase chlorophyll biosynthesis and photosynthesis, strengthen stress tolerance, and improve crop quality and yield.

The earth contains 92 elements, of which 82 can be found in plants (Reimann et al., 2001). Ti contents in plants range from 1 to 578 mg kg^−1^ with a mean of 33.4 mg kg^−1^ across the listed species (Table 1) excluding two Ti accumulators: horsetail (*Equisetum* spp.) and beach morning glory [*Ipomoea pes-caprae* (L.) R. Br.]. There are several factors affecting plant absorption of Ti: (1) Plant species differ in Ti uptake. Ti concentrations vary from 20 mg kg^−1^ in red cabbage (*Brassica oleracea* var. capitata f. rubra) to 1,900 mg kg^−1^ in the wood of pedunculate oak (*Quercus robur* L.) (Dumon and Ernst, 1988). Ti in horsetail ranged from 42 to 14,000 mg kg^−1^ when grown in soils rich in lead and zinc (Cannon et al., 1968). (2) Plants respond to Ti addition regardless of soil application or hydroponic culture. Increased Ti application elevates Ti concentrations in crops, such as cabbage (Hara et al., 1976), common bean (*Phaseolus vulgaris* L.) (Ram et al., 1983), corn (*Zea mays* L.) (Pais, 1983), and pepper (*Capsicum annuum* L.) (Giménez et al., 1990). Plant roots accumulate more Ti with a small amount transported to shoots (Kelemen et al., 1993). (3) Soil pH significantly affects the absorption of Ti in plants. Acid sandy soil (pH 3.1) increased Ti solubility resulting in Ti concentrations in leaves of gray hair grass (*Corynephorus canescens* P. Beauv.) and Sheep's sorrel (*Rumex acetosella* L.) up to 142 and 207 mg kg^−1^, respectively; however, leaf Ti concentrations of the same species were only 2.4 and 4.8 mg kg^−1^, respectively when grown in a soil with nearly identical total Ti concentrations but a pH at 4.9 (Ernst, 1985). Ti concentration in beach morning glory ranged from 310 to 480 mg kg^−1^ when grown in the ilmenite soil with a pH range of 7.8–7.9, whereas Ti concentration was 910 to 1,300 mg kg^−1^ in a pH range from 7.3 to 7.4 (Ramakrishna et al., 1989). (4) Foliar application is more effective for Ti absorption. Ti content in leaves and stems increased with Ti sprays but the increase was limited in soil application (Wojcik and Wojcik, 2001). Tapertip hawksbeard (*Crepis acuminata* Nutt.) is a dust-indicator plant, and seedlings of this species showed an 11-fold increase in Ti after being exposed to contaminated soil dusts (Cook et al., 2009). (5) Ti deficiency symptoms have not been described in plants. Ti supplied at low concentrations has been shown to positively affect plant growth (Figure 1) but causes phytotoxicity at high concentrations (Wallace et al., 1977).

**Table 1:** **Concentration of titanium in plants grown in soils where titanium was not applied via roots or leaves**.

**Species**	**Common name**	**Tissue**	**Mean concentrations (mg kg^−1^ DW)^z^**	**References**
*Acer rubrum* L.	Red maple	Leaves	175	Guha and Mitchell, 1966
		Stem	90	
*Acer pseudoplatanus* L.	Sycamore	Leaves	53	Guha and Mitchell, 1966
		Inflorescence	19	
		Petiole	7	
*Aesculus hippocastanum* L.	Horse chestnut	Leaves	32	Guha and Mitchell, 1966
*Alibertia concolor* Schum.	Cordiera concolor	Leaves	15	Ceccantini et al., 1997
*Allium cepa* L.	Bulb onion	Bulb	41	Connor and Shacklette, 1975
*Asparagus officinalis* L.	Garden aspargus	Stem	180	Connor and Shacklette, 1975
*Bauhinia rufa* Steud.	Bauhinia	Leaves	5	Ceccantini et al., 1997
*Beta vulgaris* L.	Red beet	Beetroot	27	Markert and Haderlie, 1996
*Betula pendula* Roth (*Betula alba*)	Silver birch or Warty birth	Leaves	6	Markert and Haderlie, 1996
*Blepharocalyx salicifolius* Berg	Maria-Black color or Murtinha	Leaves	6	Ceccantini et al., 1997
*Brassica oleracea* L.	Headed cabbage	Leaves	120	Connor and Shacklette, 1975
*Capsicum annuum* L.	Sweet pepper	Fruit	110	Markert and Haderlie, 1996
*Corynephorus canescens* (L.) P. Beauv.	Gray hair-grass	Leaves	2	Markert and Haderlie, 1996
*Citrus* L.	Species name was not given	Leaves	17	Markert and Haderlie, 1996
*Crepis acuminata* Nutt.	Tapertip hawksbeard	Leaves	40	Cook et al., 2009
*Cucumis sativus* L.	Cucumber	Fruit	19	Connor and Shacklette, 1975
*Dalbergia miscolobium* Benth.	Rosewood	Leaves	7	Ceccantini et al., 1997
*Daucus carota* subsp. sativus	Carrot	Roots	28	Connor and Shacklette, 1975
*Deschampsia flexuosaz* (L.) Trin.	Wavy hair-grass	Above ground part	2	Markert and Haderlie, 1996
*Diandrostachia chrysothrix*	Diadrostachia	Leaves	5	Ceccantini et al., 1997
*Equisetum spp*	Horesetail	Above ground part	460	Cannon et al., 1968
*Erythroxylon* spp.	Coca plant	Leaves	1	Ceccantini et al., 1997
*Fagus sylvatica* L.	Beach	Leaves	15	Guha and Mitchell, 1966
*Galium apparine* L.	Cleavers or Goosegrass	Above ground part	6	Markert and Haderlie, 1996
*Gochnatia polymorpha* Cabrera	Candeia or Cambara	Leaves	27	Ceccantini et al., 1997
*Hyloccomium splendens*	Moss	Leaves	53	Berg and Steinnes, 1997
*Ipomoea pes-caprae* (L.) R. Br.	Beach morning glory	Leaves	578	Ramakrishna et al., 1989
*Lamanonia ternata* Vell.	False piqui	Leaves	32	Ceccantini et al., 1997
*Leandra aurea* Cogn.	Leandra	Leaves	4	Ceccantini et al., 1997
*Lolium* L.	Ryegrass	Leaves	11	Markert and Haderlie, 1996
*Molinia caerulea* (L.) Moench	Purple moor-grass	Above ground part	3	Markert and Haderlie, 1996
*Orychophragmus violaceus*	Chinese violet cress	Leaves	43	Cao et al., 2014
		Inflorescence	15	
		Roots	12	
*Phaseolus vulgaris* L.	Snap bean	Green pods	72	Connor and Shacklette, 1975
*Pinus* L.	Pine	Needless	8	Markert and Haderlie, 1996
*Pinus sylvestris*	Scots pine	Needless	5	Markert and Haderlie, 1996
*Polytrichum formosum* Hedw.	Polytrichum moss	Above ground part	6	Markert and Haderlie, 1996
*Prunus serotina* Ehrh.	Black cherry	Leaves	155	Connor and Shacklette, 1975
		Stem	120	
*Pteridium aquilinum* (L.) Kuhn	Brake or Eagle fern	Leaves	20	Ceccantini et al., 1997
*Qualea grandiflora* Mart.	Brazilian savanna	Leaves	20	Ceccantini et al., 1997
*Qualea robur* L.	Pedunculate oak	Wood	1,900	Dumon and Ernst, 1988
*Rumex acetosella* L.	Sheep's sorrel or Red sorrel	Leaves	5	Markert and Haderlie, 1996
*Sphagnum* L.	Peat moss	Above ground part	10	Markert and Haderlie, 1996
*Stryphnodendron adstringens* Coville	Barbatimao	Leaves	12	Ceccantini et al., 1997
*Vaccinium angustifolium* Ait.	Lowbush blueberry	Leaves	4	Sheppard and Evenden, 1990
*Vaccinium vitisidaea* L.	Lingonberry or Cowberry	Leaves	5	Markert and Haderlie, 1996
*Vaccinium angustifolium* Ait.	Lowbush blueberry	Leaves	4	Sheppard and Evenden, 1990
*Vaccinium vitisidaea* L.	Lingonberry or Cowberry	Leaves	5	Markert and Haderlie, 1996

z *Dry weight (DW)*.

## Ti improves plant performance

The biological role of Ti in plants has been studied for more than 100 years. Pellet and Fribourg (1905) were the first to study Ti in soils and sugar cane (*Saccharum* spp.) and sugar beets (*Beta vulgaris* L.). Traetta-Mosca (1913) observed that Ti enhanced the growth of tobacco (*Nicotiana tabacum* L.) leaves and believed that Ti was an inherent constituent of the ash from all plants. They proposed that Ti might participate in plant metabolism as a redox catalyst. Geilmann (1920) found that Ti mainly accumulated in assimilation organs. A systematic study of plant responses to different concentrations of Ti by Němec and Káš (1923) showed that optimal levels of Ti caused increased plant growth and development and increased the intensity of green color (higher chlorophyll content) of mustard (*Brassica arvensis* L.), pea (*Pisum sativum* L.), and alfalfa (*Medicago sativa* L.). Subsequently, a great deal of attention from the 1920s to early 1970s has been focused on the analysis of Ti contents in wild and cultivated plants (Dumon and Ernst, 1988). Pais et al. (1977) synthesized a Ti compound called Ti-ascorbate with a trade name of Titavit. It was produced by chelating TiCl_4_ with ascorbic acid in the presence of gaseous HCl. Ti-ascorbate is water soluble, stable up to pH 8, and also not toxic to animals. Since then, Ti-ascorbate has been widely used for Ti-related plant experiments (Pais, 1983; Carvajal and Alcaraz, 1998; Hrubý et al., 2002; Cigler et al., 2010). A commercial product called Tytanit® containing 5% MgO, 10% SO_3_, and 0.85% other titanium complex was developed and used in central and eastern European countries for improving crop production. Ti has also been used as a beneficial element in China for crop production (Li et al., 2011).

### Effects of Ti compounds

Chelated Ti compounds applied to soils or onto leaves have been shown to increase plant biomass or crop yield (Table 2). Foliar spray of water-soluble Ti at 1 mg L^−1^ led to a 20% increase of dry matter of common bean (Ram et al., 1983). Application of 0.04% Ti increased total yield of wandflower (*Sparaxis tricolor* Ker. Gawl.) corms by 20% and commercial yield by 7% (Marcinek and Hetman, 2008). Kleiber and Markiewicz (2013) investigated Ti effects on tomato plants (*Solanum lycopersicum* L.) and reported that soil addition of 960 g Ti ha^−1^ for 1 year increased the yield of fruits, but had no significant effects on dry matter and sugars in fruits. Ti addition increased height of some annual bedding plants (Whitted-Haag et al., 2014). Different tissue dry weights of apple trees (*Malus pumila* Mill.) grown in the Brzenza region of Poland increased after Ti fertilization (Wojcik and Wojcik, 2001). Pais (1983) summarized Ti experiments conducted from 1974 to 1983 in Hungary and found that more than 90% of the described experiments showed yield increase ranging from 10 to 20% in different crops.

**Table 2:** **Effects of titanium compounds applied via roots or leaves on plant performance**.

**Plant species**	**Ti application**	**Beneficial effects**	**References**
*Anacystis nidulans* Drouet and Daily (Blue-green algae)	Treated with 10^−8^ *M* Ti-ascorbate	Increased biomass production, enhanced photosynthetic oxygen evolution and fructose-1,6-bisphosphatease activity	Kiss et al., 1985
*Antirrhinum majus* L. (Snapdragon)	Foliar application of 0–100 mg L^−1^ Ti-ascorbate	Increased plant height and leaf number	Whitted-Haag et al., 2014
*Avena sativa* L. (Oats)	Ti-ascorbate used in a hydroponic experiment with Ti in 0–18 mg L^−1^	Increased tissue Fe and Mg contents, stimulated nitrate reductase activity, and enhanced chlorophyll a and b contents	Hrubý et al., 2002
*Brassica oleracea* L. (Cabbage)	Foliar spray of a chelated-Ti solution at 2 mg L^−1^	Increased yield by an average of 15.7%	Pais, 1983
*Capsicum annuum* L. (Pepper)	Foliar spray of a 2 mg Ti L^−1^ solution at 35 ml per plant	Increased biomass production	Lopez-Moreno et al., 1995
*Capsicum annuum* L.	Foliar application of 0.042 mM Ti-ascorbate	Enhanced the activity of Fe-dependent enzymes	Carvajal et al., 1994
*Capsicum annuum* L.	Foliar application of 2 mg L^−1^ Ti-ascorbate	Increased fruit quality	Martinez-Sanchez et al., 1993
*Capsicum annuum* L.	Foliar application of 0.042 mM Ti-ascorbate	Improved N uptake	Frutos et al., 1996
*Capsicum annuum* L. (Paprika pepper)	Foliar spray of chelated-Ti solutions 3 and 6 mg L^−1^ three times	Yield increased from 32 to 95.3%	Pais, 1983
*Fragaria x ananassa* Duchesne (Strawberry)	Foliar application of 0.02% Tytanit	Increased total anthocyanin content	Skupień and Oszmiański, 2007
*Malus pumila* Mill. (Apple)	Foliar application of 2 g Ti ha^−1^	Improved plant growth vigor	Wojcik, 2002
*Malus domestica* L. (Jonathan-apple)	Foliar spray of a chelated-Ti solution at 3 mg L^−1^ three times	Increased yield by 16.6%	Pais, 1983
*Malus domestica* L.	Foliar application of Ti-ascorbate	Increased crop yield	István et al., 1991
*Malus pumila* Mill.	Foliar spray of 0.5 mg Ti (TiCl_4_) per plant	Increased biomass and the uptake of P, Fe, Mn, and Zn, and enhanced chlorophyll biosynthesis	Wojcik and Wojcik, 2001
*Pelargonium* x *hortorum* (Geranium)	Foliar application of 0–100 mg L^−1^ Ti-ascorbate	Increased plant growth and quality	Whitted-Haag et al., 2014
*Petroselinum crispum* Fuss (Parsley)	Foliar spray of a chelated-Ti solution at 5 mg L^−1^	Increased yield by 18.3%, and reduced P deficiency	Pais, 1983
*Phaseolus vulgaris* L. (Bean)	Foliar application of Ti (TiCl_4_) at 0–1 mg L^−1^	Increased chlorophyll contents and crop yield	Ram et al., 1983
*Phleum pratense* L. (Timothy grass)	Foliar application of 0.2–0.8 L of Tytanit per hectare	Increased seed yield, thousand grain weight, and seed germination	Radkowski et al., 2015
*Pisum sativum* L. (Green-pea)	Foliar application of Ti-ascorbate	Increased the uptake of essential elements and crop yield	István et al., 1991
*Prunus domestica* L. (Plum)	Foliar spray of 0.042 mM Ti-ascorbate at 5 L per tree	Improved plant growth and increased Ca, Fe, Cu, and Zn concentrations in peel and flesh	Alcaraz-Lopez et al., 2003
*Prunus persica* var. nectarine (Nectarine)	Foliar application of 0.042 mM Ti^4+^	Extended the storability of fruits	Serrano et al., 2004
*Prunus persica* (L.) Batsch (Peach)	Foliar spray of a chelated-Ti solution at 1 mg L^−1^	Increased yield by 22.1%	Pais, 1983
*Prunus persica* L.	Foliar application of 0.042 mM Ti^4+^	Extended the storability of fruits	Serrano et al., 2004
*Ribes uva-crispa* L. (Gooseberry)	Foliar spray of a chelated-Ti solution at 1 mg L^−1^	Increased yield by 19.8%	Pais, 1983
*Rubus idaeus* L. (Raspberry)	Foliar application of 0.04–0.1% Tytanit	Increased yield and fruits quality	Grajkowski and Ochmian, 2007
*Solanum lycopersicum* L. (Tomato)	Foliar spray of a chelated-Ti solution at 5 mg L^−1^	Fruit weight increased from 11% to 25%	Pais, 1983
*Solanum lycopersicum* L.	Foliar spray of a chelated-Ti solution at 5 mg L^−1^	Fruit weight increased from 11% to 25%	Pais, 1983
*Solanum lycopersicum* L.	Tytanit dissolved in nutrient solutions with Ti equivalent to 0–960 g Ti·ha^−1^ yr^−1^	Increased yield, improved fruits quality including vitamin C content, and promoted macronutrient uptake	Kleiber and Markiewicz, 2013
*Solanum lycopersicum* L.	A hydroponic culture containing 1–2 mg L^−1^ Ti	Improved plant growth when N in nutrient solutions was low	Haghighi et al., 2012
*Solanum lycopersicum* L.	Treatment of plants with Ti concentrations from 0 to 60 10^−5^M	Increased the activity of lipoxygenase	Daood et al., 1988
*Solanum lycopersicum* L.	Tytanit dissolved in nutrient solutions with Ti equivalent to 0–960 g Ti·ha^−1^ yr^−1^	Increased Fe, Mn, and Zn uptake and lycopene content.	Markiewicz and Kleiber, 2014
*Solanum tuberosum* L. (Potato)	Foliar spray of a 2 mg L^−1^ chelated Ti solution	Increased yield by 10.2%	Ram et al., 1983
*Sparaxis tricolor* Ker Gawl. (Wandflower)	Foliar application of 0.02–0.08% Tytanit	Increased yield and essential element uptake	Marcinek and Hetman, 2008
*Triticum aestivum* L. (Wheat)	Foliar application of Ti-ascorbate	Increased crop yield	István et al., 1991
*Triticum aestivum* L.	Ti-ascorbate (5 mg L^−1^) in hydroponic solutions	Reduced heavy metal damage	Leskó et al., 2002
*Triticum aestivum* L	Foliar application of Mg- Titanit	Increased chlorophyll content and crop yield	Kovacik et al., 2014

Plant biomass or crop yield increase has been attributed to Ti-enhanced chlorophyll biosynthesis and enzymatic activities and increased photosynthesis and nutrient uptake (Dumon and Ernst, 1988; Cigler et al., 2010). Ti application increased the concentration of chlorophyll a and b as well as total chlorophyll in common bean (Ram et al., 1983), wheat (*Triticum aestivum* L.) (Kovacik et al., 2014), and other plant species (Traetta-Mosca, 1913; Bottini, 1964; Pais et al., 1969, 1977). Ti enhanced photosynthetic oxygen evolution and generated a three-fold increase of fructose-1,6-biphosphatase in blue green algae (*Anacystis nidulans* Drouet and Daily) (Kiss et al., 1985). Ti stimulates the activity of nitrate reductase in common bean (Nautsch-Laufer, 1974). Catalase was activated by Ti-ascorbate and TiCl_4_ at all development stages of embryos, seeds, and seedlings of red pepper (*Capsicum annuum* L.) (Carvajal et al., 1994). Lipoxygenase (Daood et al., 1988) and phosphofructokinase activities (Simon et al., 1988) were enhanced in tomato plants after Ti addition. Ti application also boosted plants' abilities to take up other nutrients. The contents of N, P, Ca, and Mg of greenhouse-grown tomato plants increased after Ti application (Kleiber and Markiewicz, 2013). Leaves of paprika pepper (*Capsicum annuum* L.) sprayed with Ti-ascorbate showed a significant increase of Fe and Ti concentrations (Carvajal et al., 1995).

Application of Ti can also improve crop quality. Spice red pepper (*Capsicum annuum* L. cv. Mihalyteleki) treated with Ti-ascorbate showed increased concentrations of β-carotene and xanthophylls; capsanthin content also increased 1.4 times as a function of Ti addition (Biacs et al., 1997). Tomato plants grown on rockwool supplied with a nutrient solution containing Ti equivalent to 80 g per hectare a year had elevated levels of vitamin C and total sugar in the fruits (Kleiber and Markiewicz, 2013). Foliar spray of Ti increased vitamin C biosynthesis in fruits of peppers (Martinez-Sanchez et al., 1993). Ti application also increased vitamin C contents in six cultivars of strawberries (*Fragaria* x *ananassa* Duch.) and anthocyanin contents in three cultivars (Skupień and Oszmiański, 2007). Fruit soluble solids, firmness and size of three primocane raspberry (*Rubus idaeus* L.) cultivars increased after the fruits were sprayed with Tytanit before harvest (Grajkowski and Ochmian, 2007). Pre-harvest spraying of a solution containing 0.1 mM Ca^2+^, 0.103 mM Mg^2+^, or 0.042 mM Ti^4+^ to peaches (*Prunus persica* L.) and nectarines (*Prunus persica* L., Batsch, var. *nucipersica*) improved fruit color, ripening index and firmness at harvest (Serrano et al., 2004). Peach fruit weight and firmness significantly increased, and weight loss during storage significantly decreased after foliar application of Ti, or Ti with Ca and/or Mg before harvest (Alcaraz-Lopez et al., 2004a,b).

### Effects of TiO_2_NPs

There has been an increasing amount of attention in the literature regarding effects of TiO_2_NPs on plant performance (Tables 3, 4). TiO_2_NPs have been studied for influence on seed germination. Seeds treated with TiO_2_NPs suspensions exhibited increased germination rates, enhanced root lengths or improved seedling growth of *Arabidopsis thaliana* (L.) Heynh. (Szymanska et al., 2016), cabbage (Andersen et al., 2016), oilseed rape or canola (*Brassica napus* L.) (Mahmoodzadeh et al., 2013), corn (Andersen et al., 2016), cucumber (Servin et al., 2012), fennel (*Foeniculum vulgare* Mill.) (Feizi et al., 2013), lettuce (*Lactuca sativa* L.) (Andersen et al., 2016), oat (*Avena sativa* L.) (Andersen et al., 2016), onion (*Allium cepa* L.) (Haghighi and Teixeira da Silva, 2014), parsley (*Petroselinum crispum* Mill.) (Dehkourdi and Mosavi, 2013), red clover (*Trifolium pretense* L.) (Gogos et al., 2016), soybean (*Glycine max* Merr.) (Rezaei et al., 2015), spinach (*Spinacia oleracea* L.) (Zheng et al., 2005), tomato (Haghighi and Teixeira da Silva, 2014), and wheat (Feizi et al., 2012; Mahmoodzadeh and Aghili, 2014; Gogos et al., 2016). Application of TiO_2_NPs increased plant tolerance to abiotic and biotic stresses, including cold stress in chickpea (*Cicer arietinum* L.) (Mohammadi et al., 2013, 2014), heat stress in tomato (Qi et al., 2013), drought in wheat (Jaberzadeh et al., 2013) and flax (*Linum usitatissium* L.) (Aghdam et al., 2016), cadmium toxicity in green algae (*Chlamydomonas reinhardtii* P.A. Dang) and soybean (Yang et al., 2012; Singh and Lee, 2016), and bacterial spot disease caused by *Xanthomonas perforans* in tomato (Paret et al., 2013). Foliar spray of TiO_2_NPs increased chlorophyll content in tomato (Raliya et al., 2015a) and oilseed rape (Li et al., 2015), enhanced the activity of Rubisco (Ribulose-1,5-bisphosphate carboxylase/oxygenase), and promoted net photosynthesis in *Arabidopsis* (Ze et al., 2011), spinach (Hong et al., 2005a,b; Lei et al., 2007, 2008), tomato (Qi et al., 2013), and basil (*Ocimum basilicum* L.) (Kiapour et al., 2015). TiO_2_NPs treatments significantly increased crop yield or biomass of barley (Moaveni et al., 2011), corn (Moaveni and Kheiri, 2011; Morteza et al., 2013), mung bean (*Vigna radiate* L.), snail clover (*Medicago scutellata* Mil.), tomato (Raliya et al., 2015a,b), and wheat (Rafique et al., 2015).

**Table 3:** **Beneficial effects of titanium dioxide nanoparticles (TiO_2_NPs) on seed germination and plant growth**.

**Plant species**	**Application method**	**Beneficial effects**	**References**
*Allium cepa* L. (Onion)	Seeds treated with nanoparticle solutions (0, 100, 200, and 400 mg L^−1^)	Promoted seed germination	Haghighi and Teixeira da Silva, 2014
*Allium cepa* L.	Seeds treated with nanoparticle solutions (0, 250, 500, and 1,000 μg mL^−1^)	Increased seedling root growth	Andersen et al., 2016
*Alyssum homolocarpum* Fisch. Et Mey. (Qudume shirazi)	Seeds soaked with nanoparticle solutions (0, 10, 20, 40, and 80 mg.L^−1^)	Enhanced seed germination	Hatami et al., 2014
*Arabidopsis thaliana* (L.) Heynh. (Mouseear cress)	Seeds were immersed in 100, 250, 500, and 1,000 mg.L^−1^ nanoparticle solutions	Enhanced root growth	Szymanska et al., 2016
*Avena sativa* L. (Oats)	Seeds treated with nanoparticle solutions (0, 250, 500, and 1,000 μg mL^−1^)	Promoted seed germination and seedling root growth	Andersen et al., 2016
*Brassica napus* L. (Canola)	Seeds treated with nanoparticle solutions (0, 10, 100, 1,000, 1,200, 1,500, 1,700, and 2,000 mg L^−1^)	Promoted seed germination and seedling growth	Mahmoodzadeh et al., 2013
*Brassica oleracea* L. (Cabbage)	Seeds soaked with nanoparticle solutions (0, 250, 500, and 1,000 μg L^−1^)	Promoted seed germination and root growth	Andersen et al., 2016
*Chlamydomonas reinhardtii* P.A. Dang (Green algae)	Alga treated with nanoparticle solutions (0, 1, 3, 10, 30, and 100 mg L^−1^)	Reduced Cd toxicity	Yang et al., 2012
*Cicer arietinum* L. (Chickpea)	Foliar spray of nanoparticle (0, 2, 5, and 10 mg L^−1^)	Increased cold tolerance	Mohammadi et al., 2013
*Cicer arietinum* L.	Foliar spray of nanoparticle (0, 2, 5, and 10 mg L^−1^)	Increased cold tolerance	Mohammadi et al., 2014
*Cucumis sativus* L. (Cucumber)	Seeds treated with nanoparticle solutions (0–4,000 mg L^−1^)	Increased root length	Servin et al., 2012
*Cucumis sativus* L.	Seeds treated with nanoparticle solutions (0, 250, 500, and 1,000 μg mL^−1^)	Promoted seed germination and seedling root growth	Andersen et al., 2016
*Foeniculum vulgare* Mill. (Fennel)	Seeds treated with nanoparticle solutions (0, 5, 20, 40, 60, and 80 mg L^−1^)	Enhanced seed germination and seedling growth	Feizi et al., 2013
*Glycine max* Merr. (Soybean)	Foliar spray of nanoparticle (0, 0.01, 0.03, and 0.05%)	Increased crop seed yield and oil content	Rezaei et al., 2015
*Glycine max* Merr.	Seeds treated with nanoparticle solutions (0, 250, 500, and 1,000 μg mL^−1^)	Promoted seed germination	Andersen et al., 2016
*Glycine max* Merr.	Soil application of nanoparticle solutions (0–300 mg kg^−1^)	Increased Cd uptake and minimized Cd stress	Singh and Lee, 2016
*Hordeum vulgare* L. (Barley)	Nanoparticle added to MS medium (0, 10, 30, and 60 mg.L^−1^)	Increased callugenesis and the size of calli.	Mandeh et al., 2012
*Hordem Vulgare* L.	Foliar spray of nanoparticle (0, 0.01, 0.02, and 0.03%)	Increased crop yield	Moaveni et al., 2011
*Lactuca sativa* L. (Lettuce)	Nanoparticle solutions (0, 25, 50, 75, and 100 mg kg^−1^) applied to a sandy loam soil	Increased P uptake and plant growth	Hanif et al., 2015
*Lactuca sativa* L.	Seeds treated with nanoparticle solution (0, 250, 500, and 1,000 μg mL^−1^)	Promoted seedling root growth	Andersen et al., 2016
*Linum usitatissimum* L. (Flax)	Foliar spray of nanoparticle solutions (0, 10, 100, and 500 mg L^−1^)	Increased drought tolerance	Aghdam et al., 2016
*Medicago Scutellata* L. (Snail medic)	Foliar spray of nanoparticle (0, 0.01, 0.02, 0.03, 0.04, and 0.06% g L^−1^)	Increased crop yield	Dolatabadi et al., 2015
*Mentha* × *piperita* L. (Peppermint)	Seeds treated with nanoparticle solutions (0, 100, 200, and 300 mg L^−1^)	Increased root length	Samadi et al., 2014
*Nigella sativa* L. (Black cumin)	Seeds soaked with nanoparticle solution (0, 10, 20, 40, and 80 mg.L^−1^)	Promoted seed germination	Hatami et al., 2014
*Ocimum basilicum* L. (Basil)	Foliar spray of nanoparticle solution (0, 0.01, and 0.03%)	Increased tolerance of drought stress	Kiapour et al., 2015
*Petroselinum crispum* (Mill.) Fuss (Parsley)	Nanoparticle added to MS medium (10, 20, 30, and 40 mg mL^−1^)	Promoted seed germination and seedling growth	Dehkourdi and Mosavi, 2013
*Raphanus sativus* L. (Radish)	Seeds treated with nanoparticle solutions (0, 100, 200, and 400 mg L^−1^)	Promoted seed germination	Haghighi and Teixeira da Silva, 2014
*Salvia mirzayanii* Rech. F.& Esfand. (Salvia)	Seeds soaked with nanoparticle solutions (0, 10, 20, 40, and 80 mg.L^−1^)	Increased seed germination	Hatami et al., 2014
*Sinapis alba* L. (White mustard)	Seeds soaked with nanoparticle solutions (0, 10, 20, 40, and 80 mg L^−1^)	Enhanced seed germination	Hatami et al., 2014
*Solanum lycopersicum* L. (Tomato)	Soil or foliar application of nanoparticle solutions (0–1,000 mg kg^−1^)	Improved plant growth	Raliya et al., 2015b
*Solanum lycopersicum* L.	Nanoscale TiO_2_ doped applied with zinc (500–800 mg kg^−1^)	Reduced disease	Paret et al., 2013
*Solanum lycopersicum* L.	Foliar spray of nanoparticle solutions (0, 0.05, 0.1, and 0.2 g L^−1^)	Improved photosynthesis under mild heat stress	Qi et al., 2013
*Solanum lycopersicum* L.	Seeds treated with nanoparticle solutions (0, 100, 200, and 400 mg L^−1^)	Promoted seed germination	Haghighi and Teixeira da Silva, 2014
*Spinacia oleracea* L. (Spinach)	Seeds soaked with a 0.25% nanoparticle solution, plants sprayed with a 0.25% nanoparticle solution	Enhanced the expression of Rubisco mRNA and activity of Rubisco	Xuming et al., 2008
*Spinacia oleracea* L.	Seeds soaked with a 0.25% nanoparticle solution, and plants sprayed with the same solution	Enhanced photosynthesis and improved plant growth	Lei et al., 2007
*Spinacia oleracea* L.	Seeds soaked with a 0.25% nanoparticle solution, and plants sprayed with the same solution	Decreased oxidative stress to chloroplast caused by UV-B radiation	Lei et al., 2008
*Spinacia oleracea* L.	Seeds soaked with a 0.03% nanoparticle solution, and plants sprayed with the same solution	Increased activity of Rubisco activase	Gao et al., 2008
*Spinacia oleracea* L.	Seeds soaked with a 0.25% nanoparticle solution	Promoted seed germination and seedling growth	Zheng et al., 2005
*Spinacia oleracea* L.	Seeds soaked with a 0.25% nanoparticle solution, and plants sprayed with the same solution	Ti bound to the PS α reaction center complex and intensify the function of the PS α electron donor	Hong et al., 2005a
*Spinacia oleracea* L.	Seeds soaked with 0–0.6% nanoparticle solutions	Enhanced photosynthesis	Hong et al., 2005b
*Triticum aestivum* L. (Wheat)	Seeds soaked with nanoparticle solutions (0, 1, 2, 10, 100, and 500 mg L^−1^)	Promoted seed germination and seedling growth	Feizi et al., 2012
*Triticum aestivum* L.	Foliar spray of nanoparticle solutions (0.01, 0.02, and 0.03%)	Increased crop yield under drought stress	Jaberzadeh et al., 2013
*Triticum aestivum* L.	Seeds soaked with 0–1,200 mg L^−1^ nanoparticle solutions	Promoted seed germination	Mahmoodzadeh and Aghili, 2014
*Triticum aestivum* L.	Soil application of nanoparticle (0, 20, 40, 60, 80, 100 mg kg^−1^)	Improved plant growth	Rafique et al., 2015
*Triticum aestivum* L.	Seeds treated with nanoparticle solutions (0–1,000 mg L^−1^)	Promoted seedling growth	Gogos et al., 2016
*Trifolium pratense* L. (Red clover)	Seeds treated with nanoparticle solutions (0–1,000 mg L^−1^)	Promoted seedling growth	Gogos et al., 2016
*Vigna radiata* L. (Mung bean)	Foliar spray of a nanoparticle at 10 mg L^−1^	Improved crop growth	Raliya et al., 2015a
*Zea mays* L. (Maize)	Foliar spray of nanoparticle solutions (0, 0.01, and 0.03%)	Increased crop yield	Morteza et al., 2013
*Zea mays* L.	Foliar spray of nanoparticle solutions (0, 0.01, 0.02, and 0.03%)	Increased crop yield	Moaveni and Kheiri, 2011
*Zea mays* L.	Seeds treated with nanoparticle solutions (0, 250, 500, and 1,000 μg mL^−1^)	Promoted root growth of germinated seedling	Andersen et al., 2016

**Table 4:** **Negative or neutral effects of titanium dioxide nanoparticles (TiO_2_NPs) on seed germination and plant growth**.

**Plant species**	**Ti nanoparticle application**	**Effects**	**References**
*Allium cepa* L. (Onion)	Roots treated with nanoparticle solution (0, 2, 4, 6, 8, and 10 mM)	Caused DNA damages	Ghosh et al., 2010
*Arabidopsis thaliana* (L.) Heynh. (Mouseear cress)	Seedlings were grown in medium containing nanoparticles	Caused the reorganization and elimination of microtubules	Wang et al., 2011
*Arabidopsis thaliana*	Roots immersed in a 100 mg L^−1^ nanoparticle solution	No significant effects on seed germination and root elongation	Larue et al., 2011
*Brassica campestris* L. (Field mustard)	Seeds soaked with nanoparticle solutions (0, 100, 500, 1,000, 2,500, and 5,000 mg L^−1^)	No effect on seed germination	Song et al., 2013b
*Brassica napus* L. (Oilseed rape)	Roots immersed in a 100 mg.L^−1^ nanoparticle solution	No significant effects on seed germination and root growth	Larue et al., 2011
*Daucus carota* subsp. *Sativus* (Carrot)	Seeds soaked with nanoparticle solutions (0, 250, 500, and 1,000 μg L^−1^)	No effects on seed germination	Andersen et al., 2016
*Glycine max* L. (Soybean)	Plants grown in a soil mixed with nanoparticle at 0, 100 or 200 mg kg^−1^	Decreased plant growth	Burke et al., 2015
*Hordeum vulgare* L. (Barley)	Caryopses exposed to nanoparticle solutions (0, 500, 1,000, and 2,000 mg L^−1^)	No significant effects on seed germination and root elongation	Mattiello et al., 2015
*Hordeum vulgare* L.	Nanoparticles applied in a hydroponic culture (0, 100, 150, 200, 400, 600, and 1,000 mg L^−1^)	No significant effects on plant growth	Kořenková et al., 2017
*Lactuca sativa* L. (Lettuce)	Seeds soaked with nanoparticle solutions (0, 100, 500, 1,000, 2,500, and 5,000 mg L^−1^)	No effect on seed germination	Song et al., 2013b
*Lemna minor* L. (Common duckweed)	Plant growth media treated with nanoparticle (0, 10, 50, 100, 200, 1,000, and 2,000 mg L^−1^)	Inhibited plant growth	Song et al., 2012
*Lemna paucicostata* Hegelm. (Duckweed)	Nanoparticles applied to plant growth media (31, 50, and 100 mg L^−1^)	Caused growth inhibition	Kim et al., 2011
*Linum usitatissimum* L. (Flax)	Seeds treated with nanoparticle solutions (0.01–100 mg L^−1^)	High concentration inhibited seed germination, root lengths, and seedling growth	Clement et al., 2013
*Nicotiana tabacum* L. (Tobacco)	Roots treated with nanoparticle solutions (0, 2, 4, 6, 8, and 10 mM)	Caused DNA damages	Ghosh et al., 2010
*Nicotiana tabacum* L.	Seeds treated with nanoparticle solutions (0.1, 1, 2.5, and 5 %)	Decreased germination rate, root length, and seedling growth	Frazier et al., 2014
*Oryza sativa* L. (Rice)	Seeds soaked with nanoparticle solutions (100, 500, and 1,000 mg L^−1^)	No significant effects on seed germination	Boonyanitipong et al., 2011
*Solanum esculentum* L. (Tomato)	Seeds soaked with nanoparticle solutions (0, 50, 100, 1,000, 2,500, and 5,000 mg L^−1^)	Reduced seed germination and seedling growth	Song et al., 2013a
*Trifolium pratense* var. Merula (Red clover)	Nanoparticles applied in a hydroponic solution	Decreased plant growth	Moll et al., 2016
*Triticum aestivum* L. (Wheat)	Plants grown in a soil mixed with nanoparticle (10 g nanoparticle mixed with 110 kg soil)	Reduced plant growth	Du et al., 2011
*Triticum aestivum* L.	Nanoparticles applied into sand medium at 100 mg L^−1^	No significant effects on plant growth	Larue et al., 2011
*Triticum aestivum* L.	Seedlings treated with a nanoparticle solution at 100 mg L^−1^	Not significantly	Larue et al., 2012a
*Ulmus elongate* L.K. Fu& C.S. Ding (Long raceme elm)	Foliar application of 0.1, 0.2, and 0.4% nanoparticle solutions	Reduced photosynthetic rate	Gao et al., 2013
*Vicia narbonensis* L. (Narbon vetch)	Seeds treated with nanoparticle solutions (0.02, 0.1, 0.2, and 0. 4%)	Reduced seed germination, root lengths, and seedling biomass	Ruffini Castiglione et al., 2010
*Zea mays* L. (Maize)	Roots immersed in nanoparticle solutions at 0.3 or 1.0 g L^−1^	Interfered with water transport	Asli and Neumann, 2009
*Zea mays* L.	Seeds treated with nanoparticle solutions (0.02, 0.1, 0.2, and 0. 4%)	Reduced seed germination, root lengths, and seedling biomass	Ruffini Castiglione et al., 2010

Application of TiO_2_NPs may not produce positive results. As presented in Table 4, some effects were neutral or negative. Less positive results could be attributed to several factors including differences in plant species, physiological status of plants at the time being evaluated, seed quality, TiO_2_NPs sizes and their uniformity, and experimental objectives and methods. For example, some experiments used TiO_2_NPs at concentrations up to 5,000 μg mL^−1^; such high concentrations may not occur naturally in the environment, and results from the studies may not provide complete information about the roles of TiO_2_NPs in plants. However, attention does need to be given to the fate and consequence of applied TiO_2_NPs within the environment and food chain (Cox et al., 2016; Tripathi et al., 2017); more thorough research in this regard should be pursued.

### Ti as a beneficial element to crop production

Results from the literature in general suggest that Ti has positive effects on plant growth and crop quality. Ti, however, is not an essential element for plant nutrition based on the criteria for essentiality (Arnon and Stout, 1939). Plants can complete their life cycle without Ti; there is no reported Ti deficiency in plants; and mechanisms of Ti action are still uncertain. As a result, Ti is considered a beneficial element proposed by Pais (1992) because it improves plant health status at low concentrations but has toxic effects at high concentrations.

As far as is known, critical tissue concentrations for Ti that are considered to be appropriate for enhancing plant growth or potentially toxic to plants have not been well determined (Huang et al., 1993; Kuzel et al., 2007). Ceccantini et al. (1997) and Tlustoš et al. (2005) stated that Ti content in plants usually varies from 0.1 to 12.0 mg kg^−1^ of dry matter. The growth of bush bean plants was not significantly different when leaf Ti contents varied from 1.2 to 11.7 mg kg^−1^ (Wallace et al., 1977). Table grape (*Vitis vinifera* L.) plants were healthy with a mean Ti content of 17.8 mg kg^−1^ in leaves (Alcaraz-Lopez et al., 2005). Oilseed rape plants grew healthily with Ti content in shoots ranging from 16.8 to 66.7 mg kg^−1^ during their flowering period (Kovacik et al., 2016). The mean Ti content in plants listed in Table 1 is 33.4 mg kg^−1^ excluding two Ti accumulators: horsetail and beach morning glory. We propose that Ti contents in leaf tissues below 15 mg kg^−1^ based on dry weight could be appropriate for plant growth. So far, limited information is available regarding critical levels of Ti in plant toxicity. Wallace et al. (1977) reported dramatic decrease in bush bean growth when Ti in leaf tissue was 202 mg kg^−1^. Kabata-Pendias and Pendias (2001) suggested that Ti content in mature leaves ranging from 50 to 200 mg kg^−1^ could be excessive or toxic. We propose that Ti contents in leaf tissues above 50 mg kg^−1^ could potentially be toxic to plants. Morphological symptoms of Ti toxicity include chlorotic and necrotic spots on leaves (Wallace et al., 1977) and reduced plant growth and crop yield.

## Mechanisms of action

Several explanations have been proposed concerning the actions of Ti as a beneficial element to plants, including (1) participation in N fixation in the nodules of legumes (Konishi and Tsuge, 1936); (2) influence on plant metabolism by increasing absorption of other nutrient elements, such as Fe and Mg (Dumon and Ernst, 1988; Simon et al., 1988); (3) involvement in redox system reactions (Ti^4+^/Ti^3+^ with Fe^3+^/Fe^2+^) thus improving the Fe activity in plant tissues (Carvajal et al., 1995) or interaction with Fe in electron transport chain and decrease of the photosystem II efficiency at a high Ti concentration (Cigler et al., 2010); (4) stimulation of enzymatic activities and photosynthesis (Carvajal and Alcaraz, 1998); and (5) hormesis (Hrubý et al., 2002; Kuzel et al., 2003). Among these claims, Ti participation in N fixation has not been documented thereafter the initial report (Konishi and Tsuge, 1936); as a result, this claim may not be valid. Hormesis is a term used by toxicologists to refer to a biphasic dose response to an environmental agent characterized by low dose stimulation or beneficial effects and a high dose inhibitory or toxic effect (Mattson, 2008). It is a biological phenomenon for almost any chemical element or drug in living things, and it cannot be considered a specific mechanism for Ti actions in plants. The other explanations are mainly focused on the physiological roles of Ti in plants and have not explored any cellular or molecular mechanisms underpinning its actions.

A common characteristic of beneficial elements is their ability to positively interact with one or more essential elements, primarily by partial substitution of essential elements: such as sodium (Na) with potassium (K), selenium (Se) with sulfur (S), cobalt (Co) with nickel (Ni), and silicon (Si) with boron (B), manganese (Mn), and phosphorus (P). Such interactions could be synergistic at a certain concentration range but may become antagonistic when the concentration is too high. For example, when K supply becomes limited in soils, Na can partially substitute for K in osmoregulation (Marschner, 2011). Both elements are alkali metals in the Group 1 column of the Periodic Table and have similar physical and chemical properties. Like K, Na can enter plant cells through K channels (Demidchik et al., 2002). Se and S are both Group VIA elements in the Periodic Table and share similar chemical properties. Se is absorbed by plants in the form of selenate through sulfate transporters (Cabannes et al., 2011). S uptake is enhanced by rhizosphere selenate; however, Se toxicity occurs if Se and S compete for a biochemical process (White et al., 2004). Co and Ni are both transition metals and are generally found together in nature. Co is synergistically related to Ni, and reports showed that toxic Co levels of 10–20 mg kg^−1^ dry mater were associated with excess Ni (Anderson et al., 1973). This is because Co and Ni share the same plasma membrane carriers (Pilon-Smits et al., 2009).

We here propose that the beneficial roles Ti plays in plants lie in its interaction with other nutrient elements, primarily Fe. This proposal is not new and has been postulated by Simon et al. (1988), Carvajal and Alcaraz (1998), and Cigler et al. (2010). More specifically, we hypothesize that Ti and Fe have synergistic and antagonistic relationships. When plants encounter Fe deficiency, Ti could induce the expression of genes related to Fe acquisition, enhancing Fe uptake and utilization and subsequently improving plant growth. Plants could have proteins that either specifically or nonspecifically bind with Ti. When Ti concentration is high in plants, it may compete with Fe for ligands or proteins. The competition could be severe, resulting in Ti phytotoxicity. As such, the beneficial effects of Ti could be particularly visible or measurable during the time when plants are near to or are experiencing Fe deficiency. This hypothesis relies on the beneficial effects of Ti that have been reviewed above and will be elaborated further in subsequent sections of this review.

Ti and Fe have similar physical and chemical properties. Both Ti and Fe are transition metals. The ionic radius and Pauling electronegativity of Ti are 0.7 Å and 1.54; the same parameters for Fe are 0.9 to 0.7 Å and 1.83. Ti and Fe occur together in nature. During magmatic processes, Ti follows Fe in magmatic crystallization. Ti^4+^ is predominantly partitioned into Fe-Ti or Fe oxides, such as ilmenite (FeTiO_3_) and magnetite (Fe_3_O_4_), or into one or more of the TiO_2_ phases, rutile (TiO_2_), and anatase (TiO_2_). The Ti-Fe-oxides and their relationships have been illustrated by triangular FeO-TiO_2_-Fe_2_O_3_ diagrams (Bowles et al., 2011). Ilmenite (FeTiO_3_) is the most widespread form of TiO_2_-bearing mineral around the world, and it provides 90% of the total world Ti. Ti has been shown to be mobile in rocks under weathering conditions and also in soils (Cornu et al., 1999). It could be possible that adaptation of plants to soils containing heavy mineral sands (derived from the weathering of ilmenite) might enable roots to absorb both Fe and Ti. Due to the abundance of Fe in soil relative to Ti and its biological functionality, more Fe is absorbed by and translocated in plants. As a result, Fe has been fulfilling much more important roles in plants. Fe is thus considered an essential element to plants, while Ti plays a complementary role, i.e., it is often found along with Fe and plays both synergistic and antagonistic roles depending on Fe concentrations in plant cells.

## Ti uptake by plants

Plant uptake of ions through roots or leaves involves both passive absorption and active transport. Passive absorption is facilitated by concentration gradients of an ion, while active transport is driven by the electrochemical gradient generated by H^+^-ATPase to allow selective ions to move across the plasma membrane through specific carriers or transporters.

### Root uptake

There has been no report about how Ti in bulk form is absorbed by roots. Plant uptake of Fe, however, has been well studied. Plant roots use two strategies for acquisition of Fe from soils: the reduction-based strategy I in non-graminaceous plants and the chelation-based strategy II in graminaceous plants (Takagi, 1976; Römheld and Marschner, 1986). In non-graminaceous plants, Fe deficiency induces the activity of ferric reduction oxidase 2 (FRO2), which results in the reduction of Fe^3+^ to Fe^2+^, and Fe^2+^ is then transported inside the root cells by an iron-regulated transporter (IRT1) located at the plasmalemma of root epidermal cells. The IRT1/FRO2 system is subjected to complex transcriptional and post-transcriptional regulations, involving Fe itself as a local inducer, and also uncharacterized systemic signals (Kobayashi and Nishizawa, 2012). In graminaceous plants, such as maize, Fe deficiency induces root secretion of deoxymugineic acid (DMA), which is synthesized from nicotianamine, a secondary amino-acid derived from methionine. DMA has a strong affinity for Fe^3+^, and the Fe^3+^-DMA chelate is transported inside the root cells by a specific transporter YS1 (yellow stripe 1). As we proposed above, the roles Ti plays in plants lie in its interaction with Fe. We hypothesize that root uptake of Ti could occur as follows: In roots of non-graminaceous plants, the applied Ti (Ti-ascorbate) could be reduced by FRO or not be reduced and could enter plant cells through the IRT1. In roots of graminaceous plants, since Ti is often applied as Ti-ascorbate, it may not be chelated with phytosiderphore, and Ti-ascorbate could directly enter cells via YS1.

Root uptake of TiO_2_NPs appears to be size selective (Tripathi et al., 2017). Larue et al. (2012a,b) proposed that threshold diameters for movement of TiO_2_NPs through root epidermis of wheat plants should be smaller than 140 nm; thresholds for transferring through parenchyma are 36 nm or less; and for passing through the Casparian band (CB), particle diameters should be strictly smaller than 36 nm. The authors further observed that TiO_2_NPs smaller than 36 nm could be transported to the stele in two ways: direct penetration of CB, this was based on the transmission electron microscopy observation that 14 nm TiO_2_NPs were inside thick CB walls of wheat roots, implying the TiO_2_NPs had crossed the CB. The other pathway is through plasmodesmata (Larue et al., 2012a,b). Additionally, TiO_2_NPs may enter plant cells through endocytosis as NPs have been shown to activate membrane receptors and induce endocytosis (Iversen et al., 2011). So far, there are no reports regarding active transport of TiO_2_NPs through either carriers or transporters as mentioned for bulk materials. Root absorption of an ultrasmall TiO_2_NP (<5 nm) was reported to be complexed with Alizarin red S nanoconjugate in *Arabidopsis* (Kurepa et al., 2010). Whether or not such a complex was absorbed through transporters or carriers is unclear.

### Leaf absorption

Ti in both bulk and nanoparticles has been applied as liquid form to above-ground plant parts, commonly known as foliar spray or foliar application (Tables 2–4). Leaf absorption initially is a nonselective and passive process driven by concentration gradients between the outside and inside of the leaf surface (Eichert and Fernandez, 2012; Fallahi and Eichert, 2013). Since foliar applied Ti is chelated with either ascorbate or citrate, it could be likely that Ti may enter the leaf apoplast through the same routes as Fe, i.e., stomata, cuticular cracks (cracks on the cuticular surface), ectodesmata, lenticels or aqueous pores (Pandey et al., 2013). After arriving in the apoplast, Ti could be transported to symplast through the active process. The mechanism by which Ti crosses cell membranes is unknown; we assume that it could be similar to root absorption of Ti through Fe transporters.

Leaf absorption of TiO_2_NPs to apoplast could be via the same paths as the bulk materials. Due to the size effects, however, small-diameter TiO_2_NPs may gain access to symplast through direct penetration. In an experiment with TiO_2_NPs, Fe_2_O_3_NPs, and MgONPs, Wang et al. (2013) found that NPs entered leaf symplast of watermelon (*Citrullus lanatus* Matsum. & Nakai) via stomata. Raliya et al. (2015a,b) studied effects of TiO_2_NPs and ZnO_2_NPs on tomato plants and reported that foliar-applied TiO_2_NPs and ZnO_2_NPS may enter leaf cells through stomata, cuticle wounds, and direct penetration.

### Seed absorption

TiO_2_NPs have been used for seed treatment. Seeds soaked in TiO_2_NPs solutions exhibited higher germination rates, increased root elongation, and improved seedling growth (Table 3). It is generally agreed that nanoparticles are able to penetrate the seed coat, resulting in increased water/nutrient absorption and improved seed germination (Hatami et al., 2014; Zhang et al., 2015; Cox et al., 2016). However, negative effects, mainly phytotoxicities, have been reported (Table 4). The negative effects could be due in part to the penetration-resultant injury. TiO_2_NPs randomly penetrate seeds. If the penetration damaged cell membranes or embryos, seed germination and subsequent growth could be adversely affected. It is worth mentioning that physiochemical properties of TiO_2_NPs rely on the NP size, morphology, and surface area (Dietz and Herth, 2011); these properties along with TiO_2_NPs concentrations are critically important for evaluation of biological materials. Some of the reported evaluations used TiO_2_NPs with variable particle sizes, and others used concentrations much higher than those commonly encountered in the environment or normally used for evaluating other nutrient elements. These may contribute to the negative effects of TiO_2_NPs on seed germination.

## Ti translocation in plants

Ti absorbed via roots or leaves is translocated to the other organs. Like most transition elements, root-absorbed Ti is largely accumulated in the roots with a small amount transported to shoots through xylem stream (Kelemen et al., 1993). Ti absorbed by leaves is translocated via phloem flow.

### Ti distribution in plants

Nautsch-Laufer (1974) was first to report the cellular distribution of Ti in plants. When corn plants were grown in a nutrient solution containing 144 mg L^−1^ Ti, 65% of cellular Ti was found in the cell wall, 27.7% in leaf cell vacuoles, and 5.1% in root cell vacuoles. Later, Kelemen et al. (1993) studied the distribution and intracellular location of Ti in wheat plants. Foliar-applied Ti was found to be unidirectionally translocated from shoots into roots, and the majority of Ti in treated cells was in a diffusible form except for those bound firmly with nuclei. Since then, there has been no report concerning the cellular distribution of bulk Ti compounds in plants.

Recently, several studies documented the distribution of TiO_2_NPs in plants. Larue et al. (2012a,b) reported that root-absorbed TiO_2_NPs with a diameter of 14 nm were translocated to entire wheat plants without modification of crystal phase. Aerosolized TiO_2_NPs with particle diameter less than 100 nm could enter leaf cells through stomata and then be distributed to stem and roots of watermelon (Wang et al., 2013). The contents of TiO_2_ in leaves, shoots, and roots of watermelon were 61.25, 33.3, and 5.45%, respectively. When TiO_2_NPs consisting of 82% anatase and 18% rutile were used for hydroponic production of cucumber, root-absorbed TiO_2_NPs were translocated to shoots (Servin et al., 2012, 2013). Ti was found in dermal cells, mesophyll, vascular systems, and trichomes of leaves as well as cucumber fruit. Ti in rutile phase was observed mainly in aerial tissues, but anatase remained in root tissues due to the size difference. Raliya et al. (2015a,b) reported that foliar applied TiO_2_NPs were transported in a bidirectional manner, and the concentration of Ti in tomato plant tissues was in an order of stem > roots > leaves > fruits.

The distribution of Ti has been documented, but how it is translocated in plants is unclear. Fe is translocated from roots to leaves by chelating with citrate through xylem vessels. Small organic molecules and various transporters, such as NRAMPs (natural resistance-associated macrophage protein) and VIT1 (vacuolar iron transporter 1), are then responsible for Fe distribution among various organs and among various subcellular compartments (Kobayashi and Nishizawa, 2012). We assume that root-absorbed Ti-ascorbate could be directly transported to leaves through xylem vessels and the transporters that facilitate Fe distribution might also be able to translocate Ti to different organs and various subcellular locations.

### Ti binding proteins

The most stable oxidation state of Ti in an aqueous oxygenated environment is Ti^4+^, which shares the ionic radius of Fe^3+^. Ti and Fe also share a thermodynamic preference for similar binding sites, though Ti^4+^ is more strongly Lewis acidic (Zierden and Valentine, 2016). In animal cells, Ti^4+^ has been shown to bind tightly to universal iron-carrier proteins (transferrins) which carried them into the tumor cell (Guo et al., 2000). Typical animal transferrins are about 80-kDa soluble proteins involved in binding, mobilizing, and delivering Fe. Tinoco and Valentine (2005) also found that *in vitro* Ti^4+^ binds more tightly than Fe^3+^ to human transferrins. A novel transferrin-like protein was identified in unicellular green alga (*Dunaliella salina* Teodor) (Fisher et al., 1997, 1998). However, such types of proteins have not yet been identified in higher plants.

The roles Ti exhibits in plants are similar to those of rare earth elements (REEs). REEs have been widely used in agriculture as plant growth stimulants (Hu et al., 2004; Tyler, 2004). Research on the roles of REEs identified a REE-binding protein in corn (Yuan et al., 2001), two from coral fern [*Dicranoptris dichotoma* (Thunb.) Dernh.] (Guo et al., 1996), and a REE-binding peptide also from coral fern (Wang et al., 2003). Recent studies showed that REEs lanthanum and terbium can activate plant endocytosis and their entrance to cells by endocytosis (Wang et al., 2014, 2016; Yang et al., 2016). REEs in soil solutions and their contents in plant tissues are much lower than Ti (Tyler, 2004). It is possible that plants may also have proteins that interact with Ti.

We hypothesize that Ti binding proteins occur in plants. Some of them could specifically bind with Ti while others may bind not only with Ti but also with Fe. Like other ions, Ti^4+^ inclines to hydrolysis and hydrolytic precipitation (Buettner and Valentine, 2012). Binding to biomolecules that are either small or large will significantly increase its solubility. As indicated by Zierden and Valentine (2016), Ti^4+^ complexes can kinetically display a wide range of ligand exchange rates. Hydroxyl and water ligands are very labile and exchange with rate constants on the order of thousands per second (Comba and Merbach, 1987); whereas the rates for exchange with small bioligands such as ascorbate or citrate, or with transferrin-like proteins transferrins are over minutes to hours (Tinoco and Valentine, 2005; Buettner et al., 2012). As such, Ti may bind with some organic acids, such as citric acid and ascorbic acid to allow the chelated Ti to be easily translocated in plants. Additionally, Fe storage protein ferritins can biomineralize Ti (Klem et al., 2008; Amos et al., 2013). Furthermore, Ti may interact with other proteins. TiO_2_NPs have been shown to bind to the PSII reaction center complex and enhance the role of the PSII electron donor (Hong et al., 2005a). A recent microarray analysis of TiO_2_NPs treated *Arabidopsis* has shown that a series of genes, particularly those associated with photosynthesis were highly upregulated (Tumburu et al., 2015), which provides some fundamental information for further investigation of Ti effects on plants. Nevertheless, we believe that Ti binding proteins could be identified with the advances in omics technologies, and the identification should provide theoretical explanations for the roles Ti plays and its phytotoxicity in plants.

## Contributions to Fe homeostasis

Plant cells contain numerous iron-containing proteins which can be mainly classified into three groups: iron-sulfur cluster proteins, hemeproteins, and non-heme/non-Fe-S proteins (Zhang, 2015). These proteins use Fe as a cofactor and perform critical roles in photosynthesis, genome stability, electron transfer, and oxidation-reduction reactions. Plants have evolved sophisticated mechanisms to maintain iron homeostasis for the assembly of functional iron-containing proteins, thereby ensuring genome stability, cell development, electron transport chain of photosynthesis and respiration in chloroplasts and mitochondria, respectively (Kobayashi and Nishizawa, 2012). Fe is also essential for reactive oxygen species (ROS) detoxification, chlorophyll biosynthesis, period length control of circadian rhythm, and activity of numerous metal-dependent enzymes (Alscher et al., 2002; Moseley et al., 2002; Chen et al., 2013). Most of the Fe in leaves is found within the chloroplasts where photosynthesis takes place to assimilate C and produce O_2_. In addition to the general mitochondrial Fe-S cluster synthesis pathway, chloroplasts are autonomous for their Fe-S cluster synthesis (Zhang, 2015). It is within this plant specific subcellular compartment that ferritins store and buffer Fe, thereby participating in remediating oxidative stress. Ferritins are plastid proteins whose abundance is strictly controlled at a transcriptional level by the Fe status of the cells (Kobayashi and Nishizawa, 2012; Zhang, 2015).

In the case of Fe and Ti interactions, Ti effects could become more pronounced when plants had deficient supply of Fe. Under such conditions, application of Ti could induce the expression of *IRT* in nongraminaceous tobacco plants and *YS1* in graminaceous corn plants. The expression of ferritin genes could also be enhanced by Ti application. The induced expression of these genes under limited Fe supply might suggest that some roles Ti would play could be the maintenance of Fe homeostasis at the cellular level, thus improving plant growth. Carvajal and Alcaraz (1995) demonstrated that foliar application of Ti-ascorbate resulted in an increase of Fe concentrations in leaves, fruits, chloroplasts, and chromoplasts of red pepper plants. Foliar application of Ti resulted in 39% and 35.7% increase of Fe in peel and flesh of peach fruit (Alcaraz-Lopez et al., 2004a,b). Leaves of paprika pepper sprayed with Ti-ascorbate increased Fe uptake by 50% in a greenhouse experiment and close to 100% in a field experiment, and leaf peroxidase and catalase activities also significantly increased due to the Ti-ascorbate application (Carvajal et al., 1995). These results provide further evidence supporting our hypothesis that the synergetic roles Ti plays become more noticeable when plants encounter low Fe supply. Under a limited Fe supply, application of an appropriate concentration of Ti would induce *IRT* or *YS1* expression, thus enhancing Fe uptake. Increased Fe uptake would increase chlorophyll biosynthesis, subsequently increasing net photosynthesis. Increased photosynthesis directly couples with ${\text{NO}}_{3}^{-}$ assimilation in chloroplasts, which is known as nitrate photoassimilation (Searles and Bloom, 2003). The increased photosynthesis would enhance the expression of nitrate transporter genes, consequently increasing N uptake. The increased uptake of ${\text{NO}}_{3}^{-}$ could improve plant growth and in turn enhance absorption of other ions. For example, a 7-fold increase in N uptake by rhododendron (*Rhododendron* spp. cv. P.J.M. Compact) was associated with a 3 to 4-fold increase in the uptake rate of phosphorus, potassium, and sulfur, and ~2-fold increase in the uptake rate of magnesium and calcium (Scagel et al., 2008). Additionally, IRT1 belongs to the ZRT/IRT-like protein (ZIP) gene family, which plays a major role in Fe/Zn (zinc) uptake (Guerinot, 2000). IRT1 can also transport Zn, Co, Mn, and cadmium (Cd) (Eide et al., 1996; Connolly et al., 2002; Varotto et al., 2002; Vert et al., 2002). YS1 functions as a proton-coupled symporter for various DMA-bound metals, including Fe^3+^, Zn^2+^, Cu^2+^, and Ni^2+^ (Kakei et al., 2012). This may explain why the application of Ti also increases plant uptake of other nutrient elements.

Ti may act antagonistically with Fe resulting in Ti toxicity in plants. If Ti concentration is too high, it could interfere with biological roles of Fe, resulting in Ti toxicity. Cigler et al. (2010) measured chlorophyll fluorescence of spinach plants after treatment by a combination of Fe and Ti. They found that Ti at a high level affects Fe-containing proteins in electron transport, primarily the PSI, slowing down the PSII efficiency. If Ti and Fe were equally present in the medium, the Ti impact on the PSI was lowered, probably due to competition for binding sites.

## Photocatalysis and antimicrobial roles

Ti in both bulk and nanoparticle forms has been used for suppressing crop diseases (Paret et al., 2013; Servin et al., 2015). Chao and Choi (2005) reported that severity and incidence of curvularia leaf spot [*Curvularia lunata* (Wakker) Boedijn] and bacterial leaf blight (*Xanthomonas oryzae* pv. oryzae) in cereal crops were reduced with TiO_2_ application. Similar results were observed on field-grown cowpea (*Vigna unguiculata* Walp.) where cercospora leaf spots caused by *Cercospora rosicola* Pass. and brown blotch caused by *Mycosphaerella cruenta* Sacc. were significantly suppressed by application of TiO_2_ (Owolade and Ogunleti, 2008). TiO_2_ has been shown to control bacterial leaf spot (*Xanthomonas hortorum* pv. pelargonii) on geranium (*Pelargonium x hortorum* L.H. Bariley) and (*Xanthomonas axonopodis* pv. poinsettiicola) on poinsettia (*Euphorbia pulcherrima* Willd. Ex klotzsch.) (Norman and Chen, 2011). Additionally, the use of TiO_2_ in recycled irrigation water was shown to eliminate both fungal and bacterial pathogens (Yao et al., 2007).

The antimicrobial roles of TiO_2_ are related to the oxidation processes even though the role of Ti-uptake resultant biological activities could not be ruled out. Recently, the photocatalytic process by UV/TiO_2_ is receiving increased attention due to the low cost and relatively high chemical stability of TiO_2_, especially in aqueous environments. It generates singlet oxygen and superoxide anion which both cause damaging cellular oxidation. Therefore, TiO_2_ has been used for controlling some bacterial and fungal pathogens in crop production (Yao et al., 2007; Owolade and Ogunleti, 2008; Norman and Chen, 2011) and also for decontaminating toxic organic pollutants in water treatment (Lazar et al., 2012). TiO_2_NPs have been shown to degrade organic pesticides and herbicides in soils via redox reactions, photocatalysis, and thermal destruction under irradiation (Mir et al., 2014; Li et al., 2016). Photocatalytic TiO_2_ has been used to kill cancer cells in human (Thevenot et al., 2008), and biomedical applications of TiO_2_NPs are promising and could play important roles for improving health care, especially cancer treatment (Yin et al., 2013).

## Conclusion

Evidence accumulated over the last 100 years suggests that Ti is relatively mobile in soils, occurs in soil solution, and is available to plants. Plants are able to absorb Ti through either roots or leaves, and Ti concentrations in plant tissues are either equal to or higher than some essential nutrient elements. Ti has been shown to improve plant performance at low concentrations. In the present article, we propose Ti and Fe have synergistic and antagonistic relationships. Ti may induce the expression of genes related to Fe acquisition, enhancing Fe uptake and utilization when plants encounter Fe deficiency. The interaction of plants with Ti as well as with Fe may result in the occurrence of Ti binding proteins in plants that either specifically bind with Ti or nonspecifically share with Fe or other elements. When Ti levels are high in plants, Ti may cause phytotoxicity. This hypothesis is not new but is updated based on the current available information. With the advances in omics technologies, we anticipate that this hypothesis will be tested and improved.

## Author contributions

All authors contributed to the acquisition and interpretation of available literature and the conception of the work. JC, SL, and XYW wrote the manuscript, and all authors revised the manuscript and approved this final version.

### Conflict of interest statement

The authors declare that the research was conducted in the absence of any commercial or financial relationships that could be construed as a potential conflict of interest.
